# Specific fluorescent signatures for body fluid identification using fluorescence spectroscopy

**DOI:** 10.1038/s41598-023-30241-7

**Published:** 2023-02-23

**Authors:** Nihad Achetib, Kim Falkena, Meghna Swayambhu, Maurice C. G. Aalders, Annemieke van Dam

**Affiliations:** 1 Department of Biomedical Engineering and Physics, Amsterdam University Medical Centers, University of Amsterdam, Meibergdreef 9, 1105 AZ Amsterdam, The Netherlands; 2 Co Van Ledden Hulsebosch Center (CLHC), University of Amsterdam, 1098 XH Amsterdam, The Netherlands; 3 Amsterdam University of Applied Science, Tafelbergweg 51, 1105 BD Amsterdam, The Netherlands; 4 Zurich Institute of Forensic Medicine, University of Zurich, Winterthurerstrasse 190/52, 8057 Zurich, Switzerland

**Keywords:** Biochemistry, Biophysics

## Abstract

Non-invasive, rapid, on-site detection and identification of body fluids is highly desired in forensic investigations. The use of fluorescence-based methods for body fluid identification, have so far remain relatively unexplored. As such, the fluorescent properties of semen, serum, urine, saliva and fingermarks over time were investigated, by means of fluorescence spectroscopy, to identify specific fluorescent signatures for body fluid identification. The samples were excited at 81 different excitation wavelengths ranging from 200 to 600 nm and for each excitation wavelength the emission was recorded between 220 and 700 nm. Subsequently, the total emitted fluorescence intensities of specific fluorescent signatures in the UV–visible range were summed and principal component analysis was performed to cluster the body fluids. Three combinations of four principal components allowed specific clustering of the body fluids, except for fingermarks. Blind testing showed that 71.4% of the unknown samples could be correctly identified. This pilot study shows that the fluorescent behavior of ageing body fluids can be used as a new non-invasive tool for body fluid identification, which can improve the current guidelines for the detection of body fluids in forensic practice and provide the robustness of methods that rely on fluorescence.

## Introduction

Body fluids often have a pivotal role in forensic investigations as they can provide valuable information on source and activity level to the forensic investigators. Body fluids are not only important for identification purposes, but also to objectively link the suspect to the crime scene, criminal act, and/or exonerate the suspect as perpetrator, with information of the type and origin of the body fluid^1^. Several presumptive and confirmatory tests have been developed, such as (bio)chemical tests, microscopic, spectroscopic methods and molecular genetics-based approaches for body fluid identification^1,2^. In general, (bio)chemical tests such as catalytic, enzymatic and immunologic based methods allow rapid on-site analysis, but are limited in specificity and sensitivity, and they are destructive to the sample^3,4^. While microscopic and molecular genetics-based approaches are methods that have a high sensitivity and specificity, these methods require time-consuming sample preparation and can only take place within a laboratory environment^1,2,4–7^. The use of spectroscopic methods is more suitable for body fluid identification as they allow rapid, non-destructive on-site analysis^8–13^. Among the spectroscopic methods, the potential of Raman spectroscopy for body fluid identification has extensively been studied^4,8–21^. Several studies have shown that Raman spectroscopy, in combination with chemometric analysis, is highly suitable for body fluid identification due to the characteristic spectral signatures of the body fluids^12,19,22^. Nevertheless, Raman spectroscopy is not yet routinely used by forensic examiners as validation studies have not been conducted and the availability of automated software for correct interpretation is lacking. Furthermore, a major disadvantage of Raman spectroscopy for forensic practice, is that the technique is not able to scan large areas to localize body fluids prior to identification. Common tools that are routinely used in casework to detect and visualize body fluids are forensic light sources. These light sources provide monochromatic light at specific wavelengths to induce intrinsic fluorescence of body fluids or to enhance the contrast between the background and body fluids^24^. The sensitivity and specificity of these tools vary widely, therefore, fluorescence detection using forensic light sources can only be used as a presumptive test and not to draw definite conclusions about the type and origin of the body fluid^23^. The use of fluorescence spectroscopy for body fluid identification has received little attention^24–26^. Most of the research that is based on the fluorescent properties of body fluids focused on the *detection* of body fluids. Moreover, since the fluorescence of body fluids changes over time, knowledge about these dynamics is beneficial for the detection of body fluids^24,27,28^. A multispectral (fluorescence) imaging system might overcome the aforementioned limitations and reveal characteristic fluorescence signatures that are specific to each body fluid. The potential use of fluorescence spectroscopy for body fluid identification has earlier been investigated for saliva^25,26^. Here, the presence of saliva on human skin was associated with the presence of an emission spectrum at 345–355 nm with excitation at 283 nm^25,26^. However, the (broad) excitation peak at 283 nm, and the 345–355 nm emission peak has also been reported in other biological traces such as fingermarks and semen^27,28^. Since fluorophores emitting fluorescence in these regions are common to all body fluids, chemometric strategies are needed to allow separation of body fluids based on subtle differences in fluorescence in the same spectral region^29,30^.

In the present study, the aim is to identify body fluid-specific fluorescent signatures of semen, saliva, serum, urine and fingermarks that can be used for body fluid identification. Furthermore, the influence of time on the fluorescent signatures of the body fluids were explored. Knowledge about the fluorescent behavior of body fluids is relevant for crime scene examiners to decide which excitation light sources should be used. The application of this knowledge in the forensic field will improve the detection of biological stains. For analysis of the fluorescence signals, principal component analysis (PCA) was performed to find the spectral region where the spectral variation between the different body fluid is the largest and to cluster the body fluids accordingly.

## Results

The aim of this study was to identify body fluid-specific fluorescent signatures of semen, serum, urine, saliva and fingermarks, which can be used for body fluid identification. To this end, excitation and emission maps (EEMs) were recorded of the different body fluids and PCA analysis was performed to find the spectral regions where the variance between body fluids is the largest. In addition, the influence of time on the fluorescent signatures of the body fluids were explored.

### Excitation and emission maps of the body fluids

**Figure 1:**
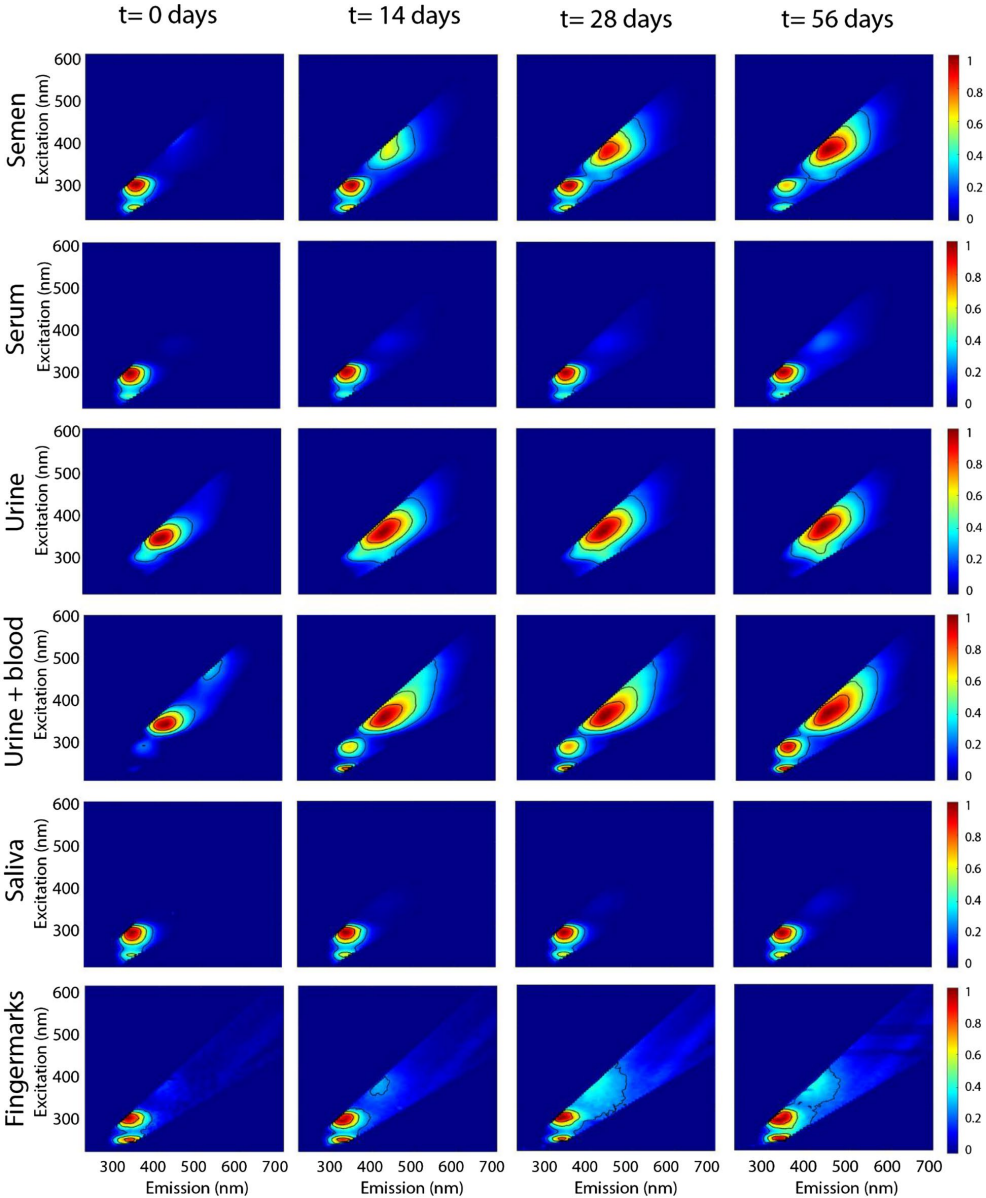
Normalized excitation and emission map of five different body fluids. The EEMs demonstrate the fluorescent properties and temporal behavior of semen, serum, urine, urine containing menstrual blood and fingermarks. In this figure the time points that were included are t = 0, t = 14, t = 28 and t = 56 days.

Typical normalized excitation and emission maps of semen, serum, urine, saliva and fingermarks are presented in Fig. 1, (Methods section, “Excitation and emission maps”). In these maps, fluorescent intensities are plotted with corresponding excitation and emission wavelength. The regions with high intensities represent the spectral location of specific fluorophores. Overall, three spectral regions were observed, except for urine. For urine, two donors which were in their menstrual period, showed these three spectral regions (see Fig. 1 for example urine + blood), while the other donors had only one broad peak, (see Fig. 1, urine). The first and second region comprises the UV excitable fluorescence with an optimal excitation-emission wavelength of ~ 225–230/335–350 nm and ~ 280–295/330–370 nm, respectively. For fingermarks these ranges were broader as the optimal excitation emission wavelength of the first and second region were at 225–230/325–365 nm and ~ 275–305/315–365 nm, respectively. The fluorescence observed at these two regions originates from aromatic amino acids. Since the position and shape of the emission spectrum of these two regions are similar, it is likely that the fluorescence originates from the same fluorophore upon excitation at different excitation wavelengths. Bortolotti et al. confirmed that emission (~ 300–350 nm) upon excitation at 220–230 nm is due to the excitation of higher excited electronic states of tyrosine and/or tryptophan, while the emission observed at excitation of 280 nm is from the lowest excited state^31^. Finally, the third region that could be identified covers the excitation and emission wavelength range from ~ 330–400/385–480 nm. However, for saliva this third region was not observed in the first three weeks.

The fluorescent intensities of the different regions changed over time. For semen and serum, the fluorescent intensities of the first and second region decreased over time, while the fluorescent intensities of the third region increased. This phenomenon has earlier been described for semen in literature^28^. With regard to urine, only the third region could be distinguished for all of the donors, showing similar to semen an increase of the fluorescent intensities over time. The urine samples containing menstrual blood also showed an increase of the fluorescent intensities of the first and second regions in contrary to semen and serum. Excitation and emission maps of saliva show that the first and second region decrease over time. As for the third region the fluorescence intensity increases during t = 28 days and t = 56 days. Finally, the second and third region in the EEMs of fingermarks show similar behavior over time as semen and serum. However, the first region did not show a clear trend as the fluorescent intensities fluctuated over time. Fingermarks contain a variation of exogenous compounds which could influence their emission spectra. Overall, the first and second region tend to decrease over time, while the third region increases. For semen and fingermarks the second region has previously been assigned to protein fluorescence and the third region to fluorescent oxidation products (FOX)^27,28^. The authors suggested that the decrease in fluorescence intensities of protein and increase of FOX fluorescence were due to oxidation process. Using the relative amounts of proteins and FOX and the rate at which these products are converted, age estimation for semen and fingermarks is possible. The protein fluorescence was measured upon excitation at 283 nm and FOX at 365 nm. These wavelengths fall within the range of the observed regions of this study and since the optimal wavelength fluctuated over time, these two wavelengths were chosen to further explore the effect of ageing of the body fluids.

### Ageing of body fluids

**Figure 2:**
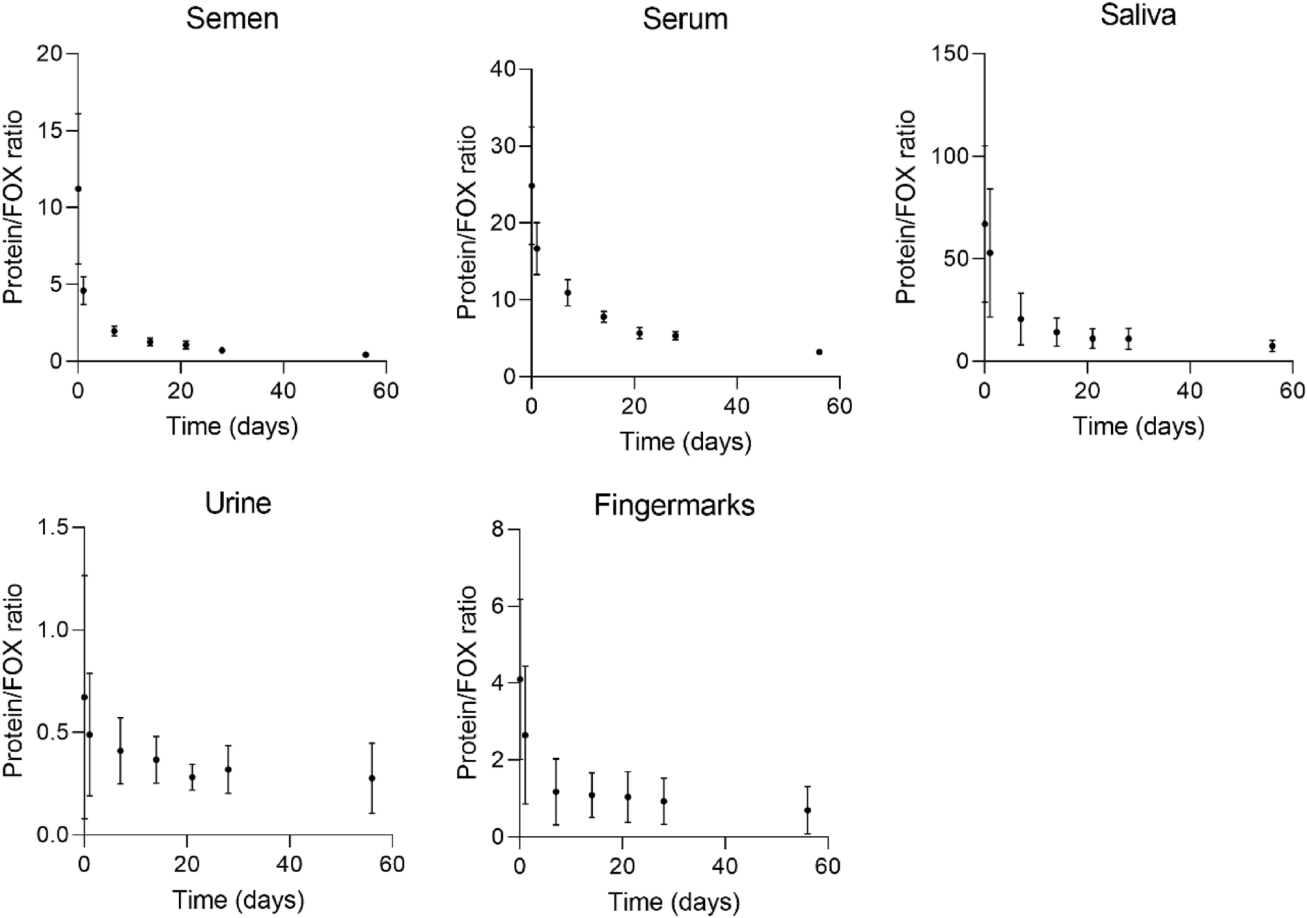
Ageing curves of the five body fluids, including semen, serum, saliva, urine and fingermarks. The ratio of the area under the curves of the Protein fluorescence at excitation 285/ emission 330–400 nm and FOX fluorescence at excitation 365/ emission 400–500 nm were plotted over time. Each dot represents the average ratio from 7 donors and the error bars represent the standard deviation.

To explore the ageing kinetics of the different body fluids, ageing curves of the Protein/ FOX ratio were constructed, by plotting the Protein/ FOX ratio against time (Methods section, “Ageing curves”). The ratio was determined by dividing the area under the curve of the protein fluorescence over the wavelength range 330–400 nm (excitation at 285 nm), by the area under the curve of the FOX fluorescence over the range of 400–500 nm (excitation at 365 nm), for each time point. Subsequently, the ageing curves of all donors were averaged for each body fluid and illustrated in Fig. 2. All body fluids showed similar decay kinetics, despite the complex chemistry of the constituents and differences in their fluorescent spectra. The ratio of intensities of the peaks at 285/330–400 nm and at 365/400–500 nm decreases over time until no further reaction kinetics could be observed. Note that especially at t = 0 the differences in ratio between the body fluids is large, while over time this difference gets smaller. For saliva, urine and fingermarks the variation in ratio among donors is larger than for semen and serum. More variation between the donor samples were expected for these body fluids, because they contain exogenous compounds from e.g. food, cosmetics or medicines, that could contribute to the Protein and FOX fluorescence. In combination with the results in section “Excitation and emission maps of the body fluids” this data proves that the position of the peak maxima and the intensities are affected by the age of the body fluid.

### Body fluid specific fluorescent signatures

**Figure 3:**
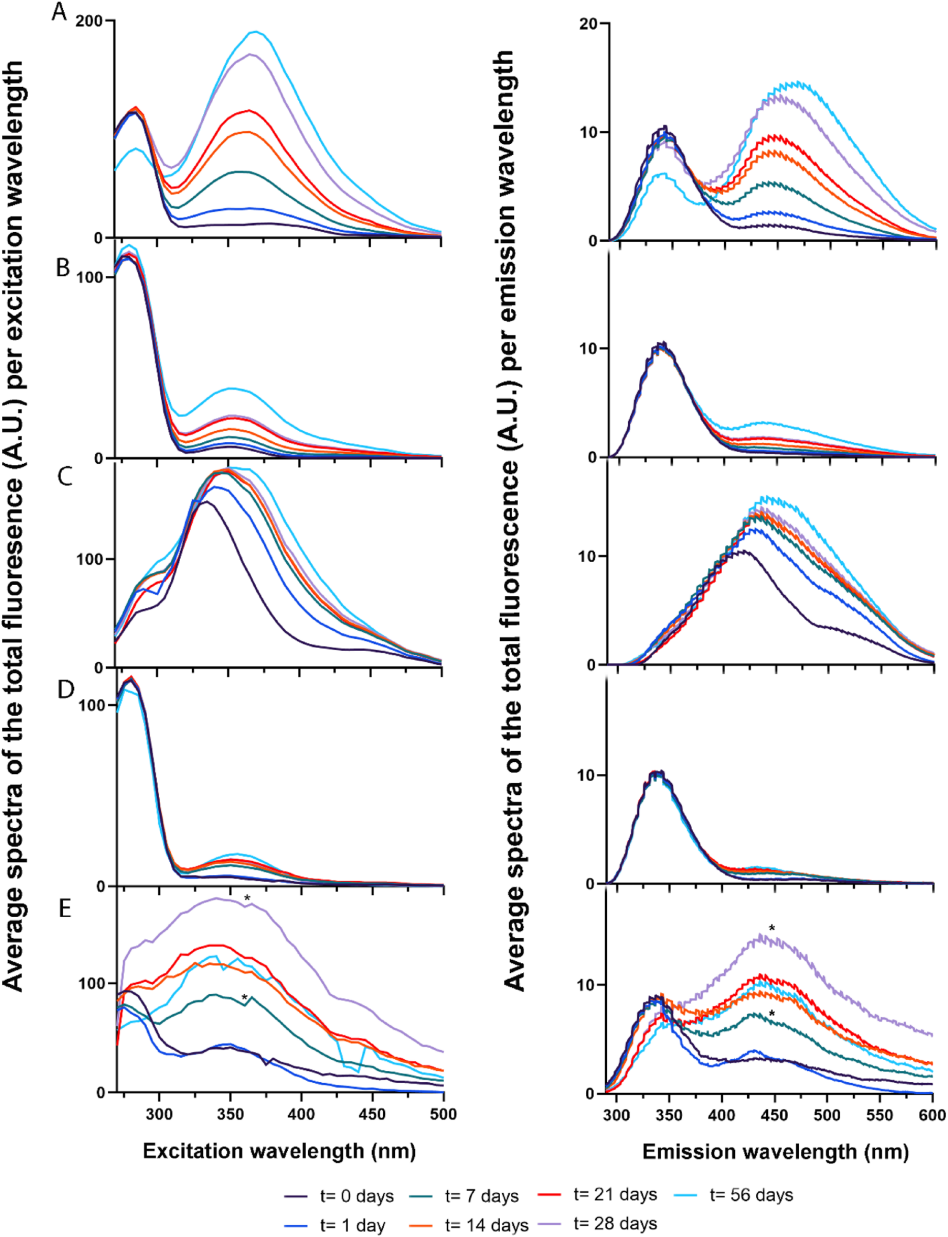
Fluorescent spectral signatures of different biological traces. The average of the sum of fluorescence per excitation (left) and emission (right) wavelengths (n = 7) for each time point for each body fluid, including semen (**A**), serum (**B**), urine (**C**), saliva (**D**) and fingermarks (**E**). Note, that the fingermarks that were 7 and 28 days old (indicated with *) were corrected with background measurements of day 1 and 21, respectively.

The average of the sum of fluorescence per excitation and emission wavelength at each time point for each body fluid (n = 7) is illustrated in Fig. 3, (Methods section, “Body fluid specific fluorescent signatures”). The errors which show the standard deviation between the different donor stains of the same age are illustrated in the Supplementary information section ‘results’, Fig. S1. Since, most of the spectral variance between body fluids was in the spectral range of 270–500 nm, analysis was only performed on the fluorescent spectra in this range, (Methods section, “Body fluid classification using principal component analysis (PCA)”). The fluorescence spectra show similarities between the body fluids, as many fluorophores are common to several body fluids. However, each body fluid exhibits an unique composition, which shows body fluid-specific fluorescent signatures which can be used to distinguish the different body fluids from each other (see Fig. 3A–E).

### Semen

For semen, the optimal excitation wavelength at all time-points are at two locations, namely at 285 nm and the second in the area between 355 and 380 nm, see Fig. 3A. The second location did not show a distinct optimal excitation wavelength over time, since the optimal excitation wavelength shifted over time (Supplementary information section ‘results’, Table S1). For the optimal emission wavelength two regions could be distinguished over time, of which the first was ~ 340–345 nm and the second region ~ 440–470 nm, (Fig. 3A).

### Serum

Serum shows two fluorescence peaks at all time-points of which the first peak has an optimal excitation wavelength at 280–285 nm and the second peak around 350–355 nm, (Fig. 3B, see supplementary information section ‘results’, table S1). The emission spectra of serum shows one major fluorescence peak, and a second peak over time. The optimal emission wavelength of the major peak at each time interval was at 342.5 nm and the second peak 436 nm, (Fig. 3B, see supplementary information section ‘results’, table S1).

### Urine

The average fluorescent excitation spectrum of urine seems to be broader than the other body fluids, with one major fluorescence peak with a peak maxima around 335–350 nm, see Fig. 3C. After one day a second peak was observed with an optimal excitation wavelength at 290 nm, however for the other time-points only a shoulder could be observed at this spectral location. A second shoulder is visible around 450 nm. The maximum fluorescence of fresh urine was recorded at 335 nm. Upon ageing a red shift of 15 nm occurred until fourteen days then the peak position did not change anymore, (Supplementary information section ‘results’, table S1). Similar to the excitation spectra, one major peak and one shoulder was observed in the emission spectra, (Fig. 3C). The optimal emission wavelength of the major peak is between ~ 420 and 445 nm. The second peak has an optimal emission wavelength at ~ 495 nm, which did not change over the course of two months, (see supplementary information section ‘results’, table S1).

### Saliva

The average excitation spectrum of saliva shows a similar pattern as serum, with the optimal excitation wavelength of the first peak at 280 nm and for the second peak around 345–355 nm (Fig. 3D). With regard to the emission, one main fluorescent peak was observed in the area of 335–345 nm which is in the area as the emission spectra of tryptophan^25^. Next, a minor peak was visible, but no distinct optimal emission wavelength (400–460 nm) could be determined.

### Fingermarks

For all time points, except at 56 days, there were two main peaks visible in the average excitation spectrum of fingermarks, (Fig. 3E). The first peak lies in the area ~ 275–290 nm and the second peak around ~ 340–480 nm. The optimal excitation wavelength of the first peak shifts towards longer wavelengths, resulting in a blue shift over time, while the second peak did not show a distinct maximum fluorescent intensity (Supplementary information section ‘results’, table S1). In the emission spectra, two peaks are observed of which the optimal emission wavelength of the first peak is at 335–365 nm and second peak at 430–440 nm, (Fig. 3E).

The spectral features of body fluids are complex as there are spectral similarities between the body fluids. Based on the literature, potential fluorophores were assigned to the observed spectral features. For semen hardly any literature has been published about the fluorescent behavior, but the excitation spectrum is reported to range from 300 to 500 nm and the emission around 460–520 nm or 400–700 nm^32^. With regard to serum, Wolfbeis et al.^33^ reported that the main fluorophores that dominate the fluorescent spectra of serum include, tryptophan (287/340 nm), NAD(P)H (345/460 nm), pyridoxic acid lactone (365/425 nm), pyridoxal phosphate Schiff base and bound bilirubin (460/515 nm). In contrary to our study, they used diluted samples, so a comparison between these studies will be difficult due to concentration variabilities. In the present study undiluted samples were used, and thus a variety of fluorescent components may be present under abundant peaks. Drzazga et al.^34^ assigned low molecular mass fluorescent compounds such as coenzymes of key enzymes in redox reactions including NAD(P)H, flavins (excitation range 330–380 nm) and fatty acids (excitation range 330–350 nm) to emit fluorescence in the visible range of undiluted serum. Similar to serum, in many studies, urine was subjected to a processing step such as diluting or centrifuging the samples. Perinchery et al.^35^ investigated why fluorescence observed in the shorter UV range (`250–300 nm) of undiluted urine is low and increases upon dilution. Their studies results showed that this occurs due to inner filter effects, concentration quenching and quenching of fluorophores by ammonium^35^. Typically body fluids encountered at a crime scene are not altered and it is therefore important to evaluate the samples in their original state. The broad excitation and emission peak of urine found in our study indicate the presence of multiple fluorophores. The excitation wavelength range of 290–460 nm, found in this study, is in agreement with previous findings reported in literature^35–37^. Fluorophores that are known to fluorescence at this excitation range included 4-pyridoxic acid, pterins, flavins and porphyrins, NADH, collagens and elastin, pyridoxic acid, uric acid, xanthine, hydroxyanthranilic acid and xanthurenic acid^35,38,39^. More studies are needed to confirm the identities of these spectral components. With regard to saliva, its fluorescence spectrum is mainly dominated by contribution of amino acids. Tryptophan, which is one of the key amino acids in α-amylase in saliva has been assigned a major contributor to the fluorescence in the region from 280 to 350 nm^25,26,40,41^. Moreover, the fluorescence observed at 400–600 nm shows similar characteristics that have been reported by Pappu et al.^42^ and Kumar et al.^43^. Pappu et al.^42^ reported that collagen, NADH, FAD and porphyrin are key fluorophores contributing to the fluorescence of saliva observed in the emission range of 400–600 nm upon excitation with 350 nm. Finally, for fingermarks, tryptophan and tryptophan derivatives including kynurenine, indoleacetic acid, norharman and xanthurenic acid have been proposed as main contributors to fingermark fluorescence^44,45^. Clearly, more studies are needed as there is a broad spectral overlap between individual fluorophores and differences in fluorophore concentration can cause shifts and spectral changes. However, by performing chemometric analysis the body fluids could be distinguished from each other, despite these overlapping spectral features.

### Body fluid classification using principal component analysis (PCA)

**Figure 4:**
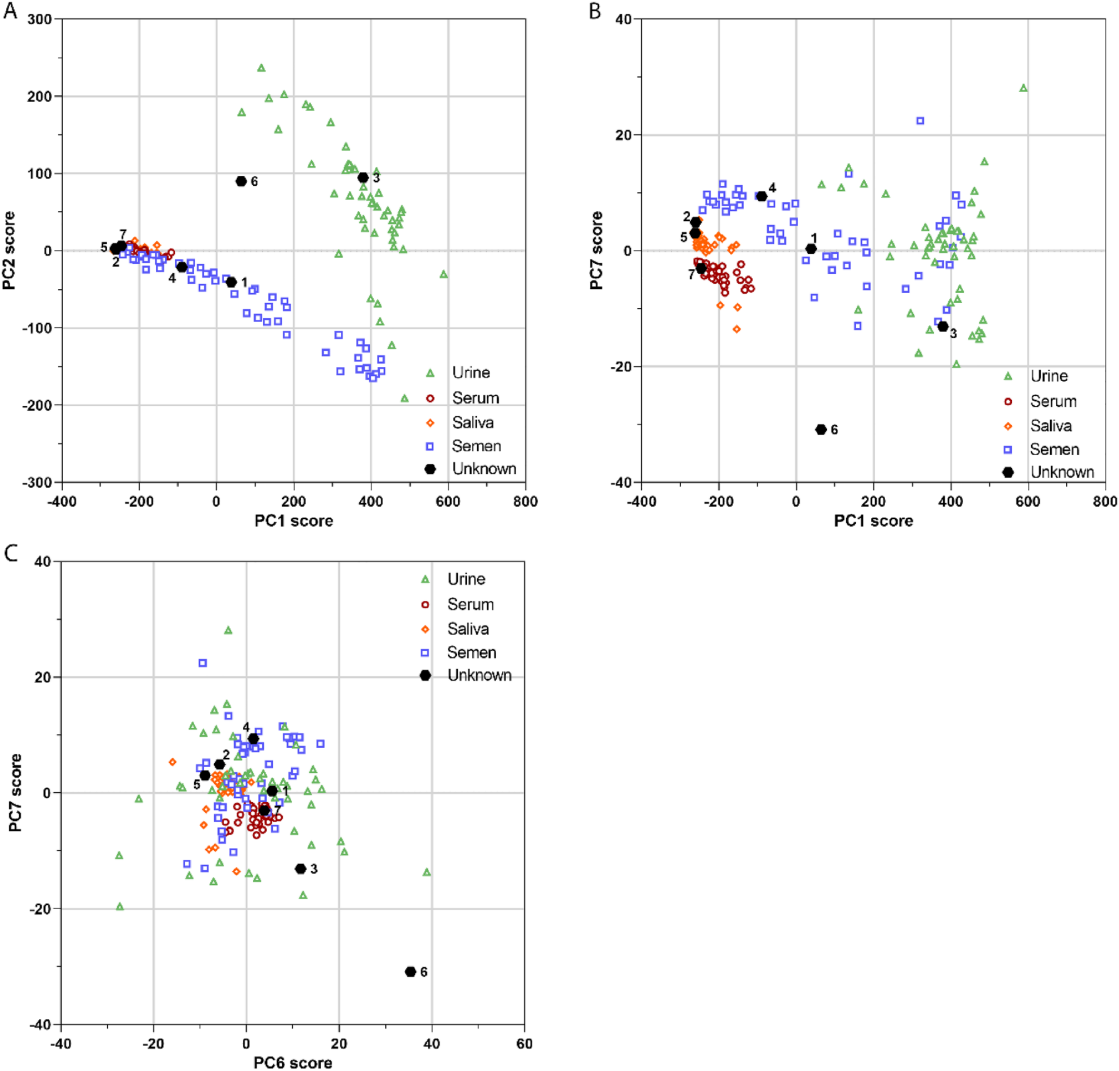
Body fluid classification using PCA. Biplots showing the scores of each sample of the principal components, including the unknown body fluid samples (n = 7, depicted as black hexagons). Each sample is plotted against the defined set of principal components, namely PC1 and PC2 (**A**), PC1 and PC7 (**B**) and PC6 and PC7 (**C**). The color and shape are depicted in the legend to represent the different body fluid types.

The EEMs illustrate the complete fluorescence characteristics of the body fluids. There are however, similarities in the spectral properties of fluorophores and overlapping regions due to the large spectral widths. Therefore, to enhance the distinctive features of the body fluids, PCA was performed using the total fluorescence of all excitation wavelengths. To reduce effects from the variance in slit widths between body fluids and to allow clustering of the unknown samples, the data were normalized. Figure 4 illustrates the PCA scores plot from semen, serum, urine and saliva. Fingermarks were excluded for PCA analysis, because their spectra showed overlap with all of the body fluids. The exclusion of the fingermarks should not be a limitation for the use of fluorescence spectroscopy for body fluid identification, since fingermarks can be recognized when ridge details of fingermark are present upon excitation with UV light.

Principal component (PC) 1 and PC2 summarized 97% of the PCA model, (Supplementary information section ‘results’, Fig. S2). In the biplot of PC1–PC2 two main clusters could be observed, see Fig. 4A. Here, urine (green) samples could be distinguished from all other body fluids, while semen (blue), saliva (black) and serum (red) samples show some overlapping scores. To distinguish semen from saliva and serum the combination of principal component PC1 against PC7 can be applied. For serum and saliva, two out of forty-nine saliva samples clustered with the serum samples. By plotting PC6 against PC7 saliva could be separated completely from serum. The combination of these principal components allow separation of the body fluids.

### Identification of the unknown body fluids using PCA analysis

To further explore the use of specific fluorescent signatures patterns as a tool for body fluid identification the fluorescence of seven body fluid samples of different ages were measured without prior knowledge of the body fluid type (Methods section, ‘Identification of the unknown body fluids using PCA analysis’). As such, the ten nearest neighbors of the seven unknowns for each PC were calculated. Figure 4 illustrate the seven unknown samples which were plotted in the original biplots. Five out of seven samples, were annotated correctly (71.4%) and two out of seven were typed inconclusive, since the classification criteria were not met (Table 1).

**Table 1:** Outcome identification unknowns using PCA.

Unknown sample #	Predicted body fluid	True body fluid	Age (days)
1	Semen	Semen	21
2	Saliva	Saliva	28
3	Urine	Urine	28
4	Semen	Semen	14
5	Saliva	Saliva	28
6	Inconclusive	Urine	28
7	Inconclusive	Serum	0

## Discussion

The current study describes for the first time body fluid specific fluorescent signatures in dried stains, which could be used for body fluid identification. The use of fluorescence in the forensic field is not new, as forensic light sources are routinely used at crime scenes to localize biological traces. However, the scientific foundation for the usage of certain wavelengths for the detection of body fluid is poor and knowledge about the fluorescent behavior of body fluids over time is unexplored. Therefore, this study provides new information on the fluorescent properties of body fluids that can improve the current guidelines. For instance, these study results show that fresh body fluids fluorescence at shorter wavelengths than aged body fluids. Furthermore, no studies explored the use of fluorescence signatures for body fluid identification (BFI) yet. This is the first study which studied the fluorescent behavior of body fluids over time to identify specific fluorescent signatures that can be used for BFI. However, optimization is required to meet the needs of the forensic community for application in the forensic field. As such, to improve the experimental set-up, the scanning time to record the fluorescence in the spectral region of 270–500 nm should be reduced. A potential strategy is to excite and measure only the spectral regions which showed the highest fluorescence intensities, an area of ~ 275–295 nm and an area of ~ 335–385 nm (Figure EEMs and figure signatures). Another strategy would be to identify the fluorophores which showed high intensities at certain spectral locations. Reducing the scanning time will allow fast, non-invasive measurements that can be performed directly at the crime scene. Wavelengths in UV region are currently not used on crime scenes to avoid risk of health and safety concerns for the examiners. However, our method uses fiber based spectroscopy, allowing local illumination of the sample. For the quality of the DNA, which can be extracted from the body fluid, we expect little impact due to the short scanning time in the UV region. Next to the optimization of the experimental set up, the data analysis can further be explored to allow a robust technique with ideally 100% accuracy for sample identification. Chemometric analysis could be extended to the use of SIMCA after PCA or other supervised classification models (e.g. LDA, PLSDA, SVMDA).

Nonetheless, more studies are needed to investigate the fluorescent behavior of these body fluids, such as increasing the sample size, include various substrates and different environmental conditions to draw definite conclusions. First of all, this research was performed under a controlled laboratory settings, however, at a crime scene the body fluid may be subjected to contaminants which may affect the fluorescence signatures. For instance for fingermarks external components such as dirt or cosmetic were found to have a large impact on the composition of fingermarks^45^. Therefore, future studies should investigate how different contaminates will influence the observed fluorescent signatures. In addition, also natural contaminates for instance, due to the dietary of the donor may contribute to the protein fluorescence of body fluids and should therefore be studied. These studies could include control experiments, whereby body fluids are spiked with tryptophan. Furthermore, knowledge about the key components that exhibit fluorescence in the different body fluids could allow excitation at single wavelengths. As these fluorophores could have similar spectral properties, the use of an additional technique to allow identification of these key components would be beneficial. Next to identification of key fluorophores, protein and lipid oxidation markers could be monitored over time to assess the hypothesis that oxidation processes play an important role in the ageing of body fluids. There is a large range of analytical methods available that could be used to identify and quantify protein and lipid oxidation markers^46–48^. Finally, this research could be extended to other biological traces such as vaginal fluid, nasal fluid, sweat and whole blood. In the current study serum samples were preferred, because hemoglobin contained or released by erythrocytes absorbs light, thereby masking the fluorescence of the fluorophores present. Furthermore, serum detection and identification may provide valuable information at the crime scene, for instance when a body is moved, whereby serum is separated from blood during clotting and cast off^49^.

Our pilot study results demonstrate the potential use of fluorescence spectroscopy for body fluid identification. Furthermore, these findings can serve as guidelines for body fluid detection by forensic examiners. More studies are needed to further optimize the method and allow application directly at a crime scene. Application of fluorescence spectroscopy in the field would be beneficial as detection and identification of body fluid could be achieved with one portable device in a non-invasive manner, while keeping the sample intact for other analyses.

## Methods

### Sample collection and preparation

Informed consents were obtained from all donors that participated in this study. The study was reviewed by the medical ethics review committee of the Academic Medical Center, whereby it was determined that the Medical Research Involving Human Subjects Act (WMO) does not apply to this specific study. Therefore, no specific study project number is available. Research protocols were performed in accordance with the Declaration of Helsinki and relevant guidelines and regulations of the Netherlands Code of Conduct for Research Integrity and the research code of the Academic Medical Center. Semen, serum, urine, saliva and fingermarks were collected from seven healthy volunteers, (see Table 2 for details of the donation procedures). The body fluids were vortexed and then two µl of donor sample for each time interval was pipetted on a TLC plate and stored in the dark at room temperature until measurement. From, each donor seven samples were prepared which were left to age for 0, 1, 7, 14, 21, 28 and 56 days. For fingermarks, each set was measured at two different locations, whereby each location represents a different time interval. The time interval between sample deposition and measurement was at least one hour for all samples at t = 0.

**Table 2:** Details of the donation procedure of semen, serum, urine, saliva and fingermarks from seven healthy donors. M presents male donors and F female donors.

Biological trace	Gender (M/F)	Place of donation	Storage (°C)	Collection procedure
Semen	7 M/0 F	Fertility clinic of Isala Hospital	− 20/− 80	Collected in sterile collection cups
Serum	3 M/4 F	Amsterdam University Medical Centers	− 80	Blood was drawn from the brachial vein into 5 ml serum blood collection tubes and centrifuged at 1885 g for 10 min
Urine	3 M/4 F	Amsterdam University Medical Centers	None	Collected in sterile collection cups. Two out of the 4 females were in their menstrual period
Saliva	2 M/5 F	Amsterdam University Medical Centers	None	Instruction to not consume any food/drinks for 1 h prior donation. A saliva collector (Oracol, Malvern Medical developments limited, UK) was used for 1 min to stimulate the saliva production
Fingermarks	4 M/ 3 F	Amsterdam University Medical Centers	None	Instruction to not wash the hands for 30 min and to avoid the use crèmes/gels. Fingermarks were rubbed together and two fingermarks were deposited on top of each other onto a TLC plate (Silicagel 60, Merck, Germany)

### Fluorescence measurements

The fluorescence of the different body fluids were measured using a fluorescence spectrometer (LS-55 Fluorescence Spectrometer, PelkinElmer Inc., USA) with a fiber accessory. The spectrofluorimeter was set at three-dimensional scan mode to record the fluorescence intensity as a function of excitation and emission wavelengths. These measurements were carried out at seven time points, namely t = 0, t = 1 day, t = 7 days, t = 14 days, t = 21 days and t = 28 days and t = 56 days. In EEM measurements, the samples were excited at 81 excitation wavelengths ranging from 200 to 600 nm, with a step increment of 5 nm for each sequential scan. For each excitation wavelength the emission was recorded between 220 and 700 nm, with a step increment of 0.5 nm increments. Since the fluorescence intensity varied between the different biological traces, the excitation and emission bandwidth were optimized to find the optimal combination of bandwidths for each type of traces. The optimized settings that were used for the measurements are listed in Table 3. For all measurements the scanning speed was set to 1500 nm/min.

**Table 3:** Spectral band pass width of excitation and emission slit.

Biological trace	Excitation slit (nm)	Emission slit (nm)
Fingermarks	5	20
Semen	10	7.5
Saliva	10	5
Urine	15	15
Serum	15	5
Unknown	10	7.5

### Excitation and emission maps (EEMs)

The excitation and emission maps were constructed and analyzed using an in-house generated Matlab script. The fluorescence spectra were first corrected for background fluorescence of the TLC plate. Since, discontinuity in the data can occur due to presence of excitation light in the emission spectra and second-order transmission, the fluorescence intensity values in these areas were set to zero. Subsequently, the data were normalized, by scaling the lowest fluorescent intensity to 0 and the highest to 1.

### Ageing curves

To construct ageing curves the area under the curves at emission wavelength intervals 330–400 nm (excitation at 285 nm) and 400–500 nm (excitation 365 nm) were calculated. The region between 330 and 400 was assigned to protein fluorescence and the region between 400 and 500 nm to fluorescent oxidation products (FOX). Subsequently, the ratio of the area under the curves of these two regions were calculated for every time point of each time series. The average ratio and standard deviation of the seven donors per body fluid were plotted in Graphpad Prism.

### Body fluid specific fluorescent signatures

**Figure 5:**
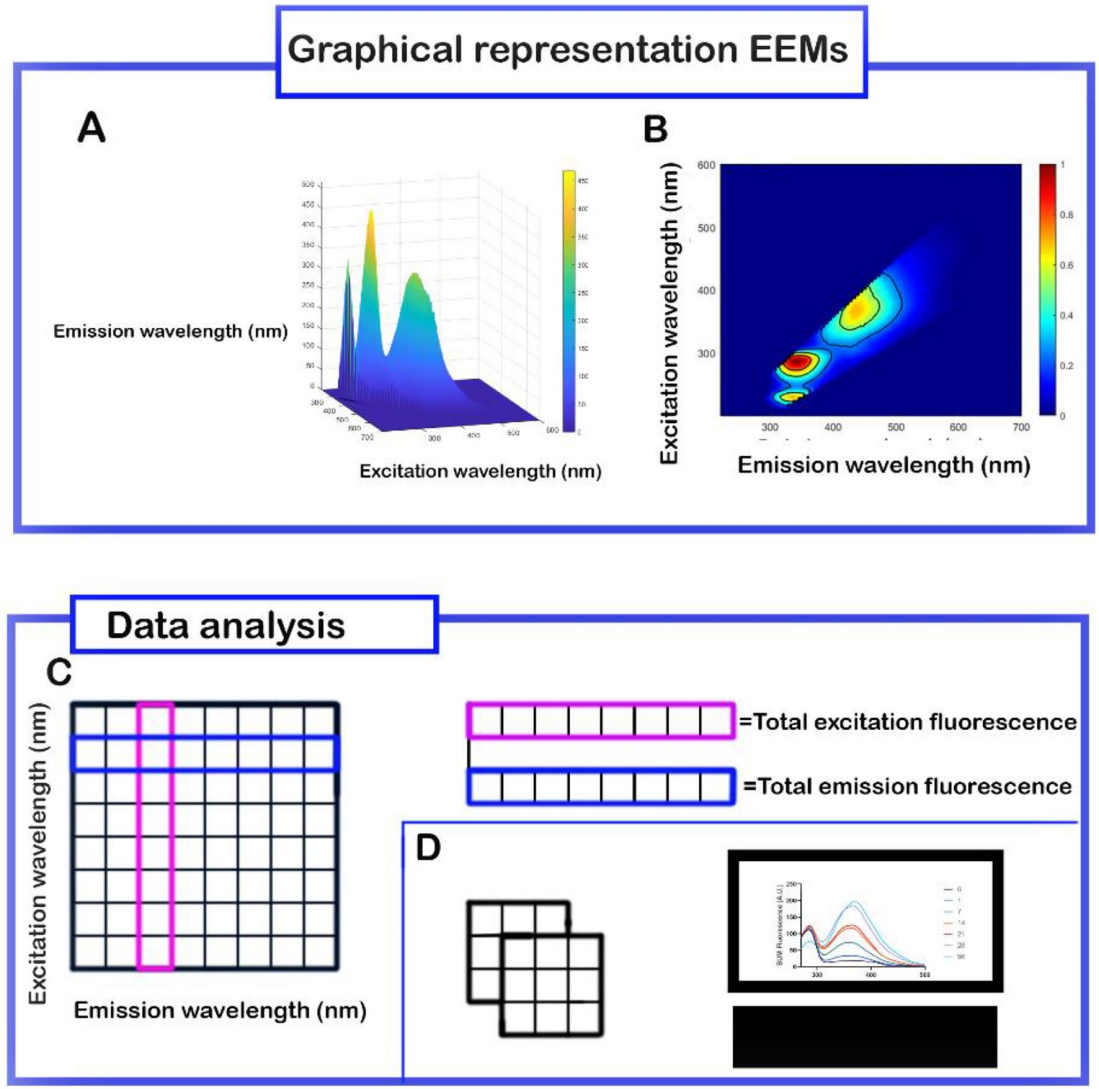
Schematic overview of data analysis to produce the body fluid signatures. (**A**) 3D view of excitation and emission map (EEMs) of a 2 weeks old semen stain. (**B**) 2D view of corrected and normalized EEMs of two weeks old semen stain. (**C**) Compute the summed fluorescence of all excitation wavelengths for each emission wavelength (pink). Compute the summed fluorescence of all emission wavelengths for each excitation wavelength (blue). (**D**) Repeat procedure for each excitation and emission wavelength for every donor on every time point, and plot the average fluorences of all donors for each time point showing the bodyfluid specific fluorescent signatures.

To demonstrate the body fluid specific fluorescent signatures for each body fluid (n = 7), the normalized fluorescent signals were summed per excitation and emission wavelength, see Fig. 5. For example, upon excitation with 200 nm, the normalized fluorescence intensity between emission wavelengths 220–700 were summed. Next to this, the total fluorescence of all excitation wavelengths for each emission wavelength were calculated. This was done individually for each donor. Subsequently, the average of the summed fluorescence per excitation and emission wavelength of seven donors were plotted to demonstrate the fluorescent pattern of each body fluid.

### Body fluid classification using principle component analysis (PCA)

PCA is a well-known multivariate chemometric method which is applied in the present study to identify spectral features that show the highest variability in the dataset. For PCA, the normalized fluorescent signals were summed per excitation and emission wavelength as has been described in the Methods section ‘Body fluid specific fluorescent signatures’. PCA analysis was performed using an in-house generated Matlab script. The data of the fingermarks were not included for the PCA. For each body fluid, samples of seven donors were measured at seven time points, resulting in a training set of 196 samples. PCA was conducted to determine where most of the spectral variance between body fluids lies in the spectral range of excitation (200–600) and emission (220–700) wavelength intervals. To this end, all analyses has been applied on the fluorescent spectra in the range of 270 to 500 nm. The principal components (PCs) were computed, of which PC1 describes most of the variance within the dataset, followed by PC2 and so on. Three combinations of four principal components were selected, which together allowed separation of all body fluids. The scores of the individual data points were plotted along the principal components in biplots.

### Identification of the unknown body fluids using PCA analysis

First, unknown samples were prepared by a researcher who was not involved in the experimental execution of this study. Fluorescence spectra were obtained as has been described in the methods section ‘Fluorescence measurements’, with one major adaption that the excitation and emission bandwidth were set to 10 nm and 7.5 nm, respectively. For one unknown (sample # 7) the fluorescent signal was saturated, thus for this particular sample, the excitation and emission bandwidth were set to 15 nm and 5 nm, respectively. After the measurements, the data of the unknown samples were arranged in a similar manner as described in the Methods section, ‘Body fluid specific fluorescent signatures’. Since the addition of the unknowns to the training set will influence the principal components (Eq. 1), a prediction score was calculated using the principal components of the training set. To eliminate differences in average amplitudes between the unknown samples and the training set, the mean amplitude was subtracted from every sample response [R]. The prediction score of the unknown sample [S] for the defined set of principal components can be derived by solving the inverse linear combination of the PCs [PCs] and the sample response of the unknown sample [R].

1 $$\left[ {{\text{PCs}}} \right]*\left[ {\text{S}} \right] \approx \left[ {\text{R}} \right]$$

The estimated scores were visualized in the original biplots and K-nearest neighbors algorithm was used to classify the unknown samples. This method is based on the distance of the unknown sample to a “k” number of samples which form clusters on spectral similarity. From each biplot, we derived the 10 nearest neighbors of the unknown sample, which resulted in a total of 30 nearest neighbors. To ensure correct annotation twenty out of thirty neighbors have at least to be from the same sample type. Samples that violate this criterion were classified as ‘inconclusive’.

## Supplementary Information

Supplementary Information.

## Data Availability

The datasets generated during and/or analysed during the current study are available from the corresponding author on reasonable request.
