# Supplementary material for: Specific fluorescent signatures for body fluid identification using fluorescence spectroscopy
Source: Sci Rep. 2023 Feb 23;13:3195. doi: 10.1038/s41598-023-30241-7 (PMC9950469; doi:10.1038/s41598-023-30241-7)
Supplement: Supplementary file 1 — Supplementary Information. [file 41598_2023_30241_MOESM1_ESM.docx]

Supplementary Information

SPECIFIC FLUORESCENT SIGNATURES FOR BODY FLUID IDENTIFICATION USING FLUORESCENCE SPECTROSCOPY

Nihad Achetib †˟, Kim Falkena †˟, Meghna Swayambhu †∞, Maurice C.G. Aalders †,‡, and Annemieke van Dam*,†°.

† Department of Biomedical Engineering and Physics, Amsterdam University Medical Centers, University of Amsterdam, Meibergdreef 9, 1105 AZ, Amsterdam, The Netherlands.
‡ Co van Ledden Hulsebosch Center (CLHC), University of Amsterdam, 1098 XH Amsterdam, The Netherlands.
 °Amsterdam University of Applied Science, Tafelbergweg 51, 1105 BD Amsterdam, The Netherlands.
∞ Zurich Institute of Forensic Medicine, University of Zurich, Winterthurerstrasse 190/52, CH- 8057, Zurich, Switzerland.
˟ Both authors contributed equally

Corresponding Author: [annemiekevandam@amsterdamumc.nl](mailto:annemiekevandam@amsterdamumc.nl)

**Abstract:** Non-invasive, rapid, on-site detection and identification of body fluids is highly desired in forensic investigations. The use of fluorescence-based methods for body fluid identification, have so far remain relatively unexplored. As such, the fluorescent properties of semen, serum, urine, saliva and fingermarks over time were investigated, by means of fluorescence spectroscopy, to identify specific fluorescent signatures for body fluid identification. The samples were excited at 81 different excitation wavelengths ranging from 200 to 600 nm and for each excitation wavelength the emission was recorded between 220-700 nm. Subsequently, the total emitted fluorescence intensities of specific fluorescent signatures in the UV-visible range were summed and principal component analysis was performed to cluster the body fluids. Three combinations of four principal components allowed specific clustering of the body fluids, except for fingermarks. Blind testing showed that 71.4 % of the unknown samples could be correctly identified. This pilot study shows that the fluorescent behavior of ageing body fluids can be used as a new non-invasive tool for body fluid identification, which can improve the current guidelines for the detection of body fluids in forensic practice and provide the robustness of methods that rely on fluorescence.

Table of Contents

1. Results

**Table S1:** Optimal excitation and emission wavelengths of different body fluids over time**S1**

**Figure S1:** Mean and standard deviation of the fluorescent spectral signatures of different biological traces **S2**

**Figure S2:** The factor loading plots**………………………………………………………………………………………………………………………...S3**

Results

**Table S1:** Optimal excitation and emission wavelengths of different body fluids over time. Some of the body fluids have multiple peaks or shoulders in their excitation and emission spectra, therefore the symbol / was used to distinguish these from each other, while the symbol – was used to show that the extra peak or shoulder was missing.

| Sample type | Age sample (days) | Optimal excitation wavelength (nm) | Optimal emission wavelength (nm) |
| --- | --- | --- | --- |
| Semen | 0 | 285/ 380 | 342.5/ 441.5 |
|  | 1 | 285/ 365 | 344/ 441.5 |
|  | 7 | 285/ 355 | 344/ 441.5 |
|  | 14 | 285/ 365 | 344/ 447 |
|  | 21 | 285/ 365 | 344/ 447 |
|  | 28 | 285/ 365 | 343.5/ 452.5 |
|  | 56 | 285/ 370 | 342.5/ 469 |
| Serum | 0 | 285/ 350 | 342.5/ - |
|  | 1 | 280/ 350 | 342.5/ - |
|  | 7 | 280/ 350 | 342.5/ - |
|  | 14 | 280/ 350 | 342.5/436 |
|  | 21 | 280/ 355 | 342.5/436 |
|  | 28 | 280/ 355 | 342.5/436 |
|  | 56 | 280/ 350 | 342.5/436 |
| Urine | 0 | -/ 335 | 419.5/ 496.5 |
|  | 1 | 290/ 340 | 430.5/ 496.5 |
|  | 7 | -/ 345 | 436/ 496.5 |
|  | 14 | -/ 350 | 436/ 496.5 |
|  | 21 | -/ 350 | 436/ 496.5 |
|  | 28 | -/ 350 | 436/ 496.5 |
|  | 56 | -/ 350 | 441.5/ 496.5 |
| Saliva | 0 | 280/ 345 | 342.5 |
|  | 1 | 280/ 350 | 342.5 |
|  | 7 | 280/ 350 | 342.5 |
|  | 14 | 280/ 350 | 342.5 |
|  | 21 | 280/ 350 | 355.5 |
|  | 28 | 280/ 350 | 342.5 |
|  | 56 | 275/ 355 | 355.5 |
| Fingermarks | 0 | 275, 350/380 | 337.5/ 431 |
|  | 1 | 275/ 350 | 337/ 430.5 |
|  | 7 | 275, 340/365 | 342.5/ 430.5 |
|  | 14 | 285, 325/365 | 342.5/ 436 |
|  | 21 | 290/ 340 | 342.5/ 436 |
|  | 28 | 290/ 340 | 359.5/ 436 |
|  | 56 | -/340/355/459 | 361/ 436 |

**
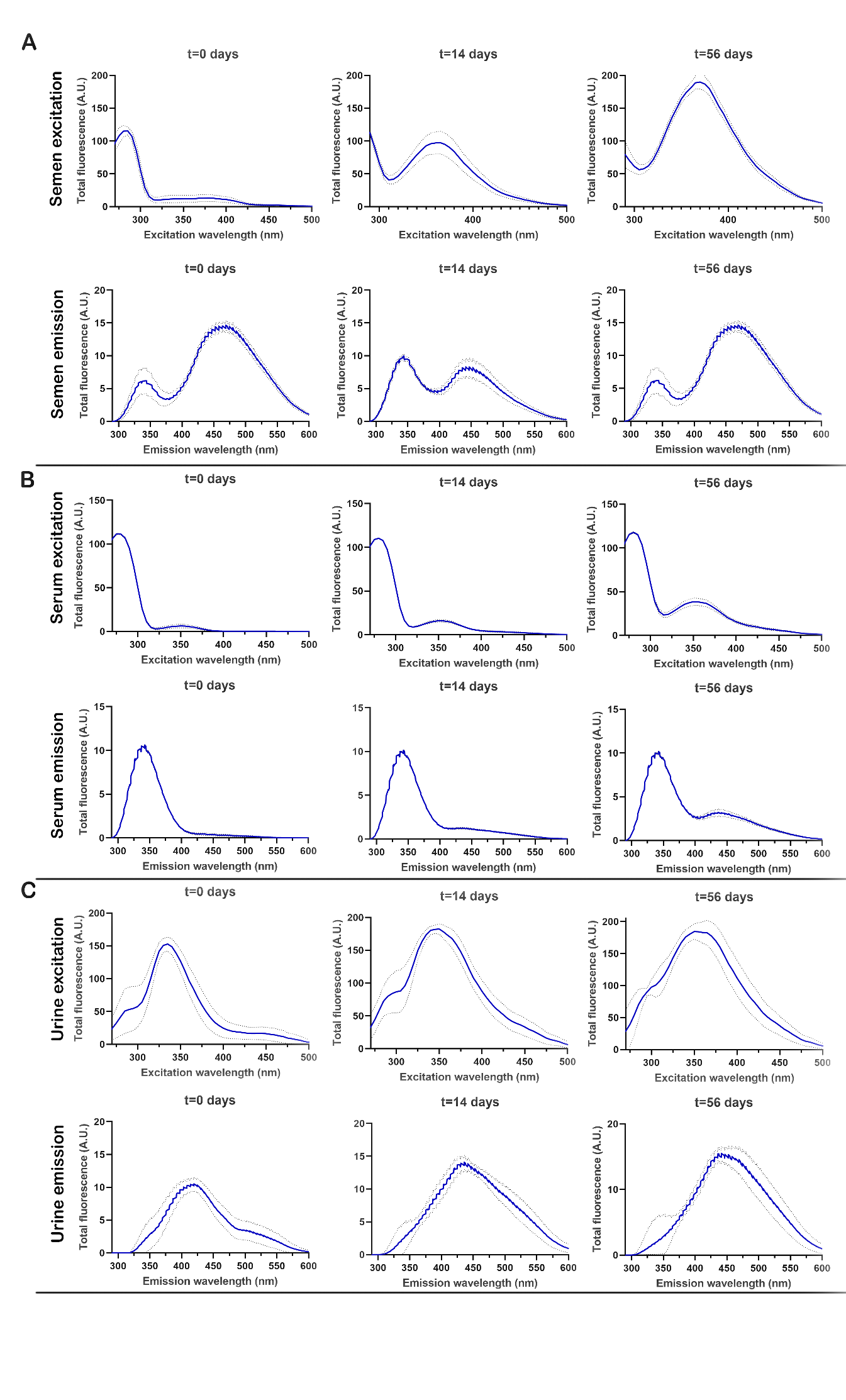
**

**
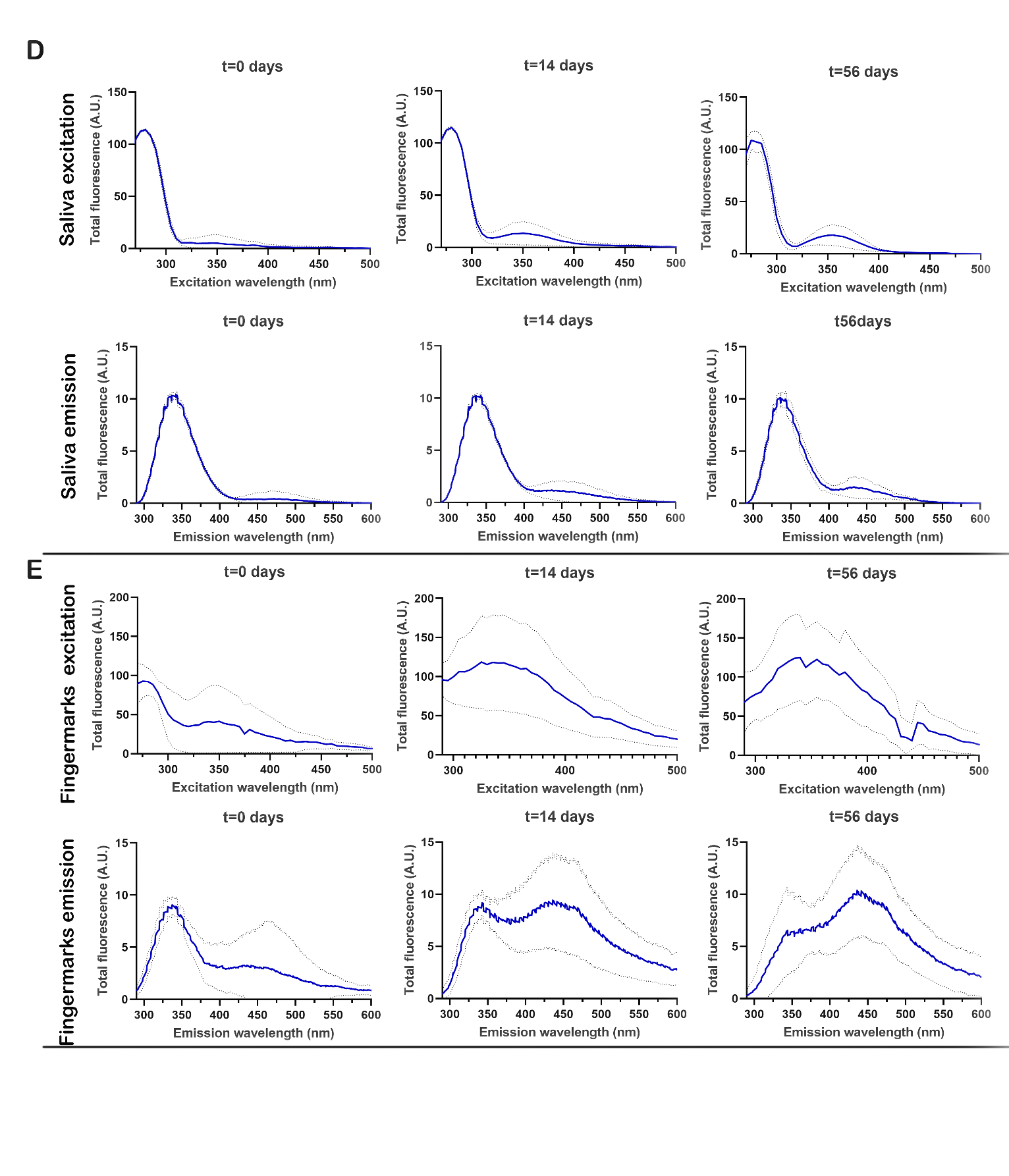
**

**Figure S1:** Mean and standard deviation of the fluorescent spectral signatures of different biological traces. The summed fluorescence per excitation (above) and emission (below) wavelengths is shown for semen (A), serum (B), urine (C), saliva (D) and fingermarks (E) on time point t= 0 days, t=14 days and t= 56 days. The blue line represent the average of the summed fluorescence of seven donors, while the gray line depicted the standard deviation.

#
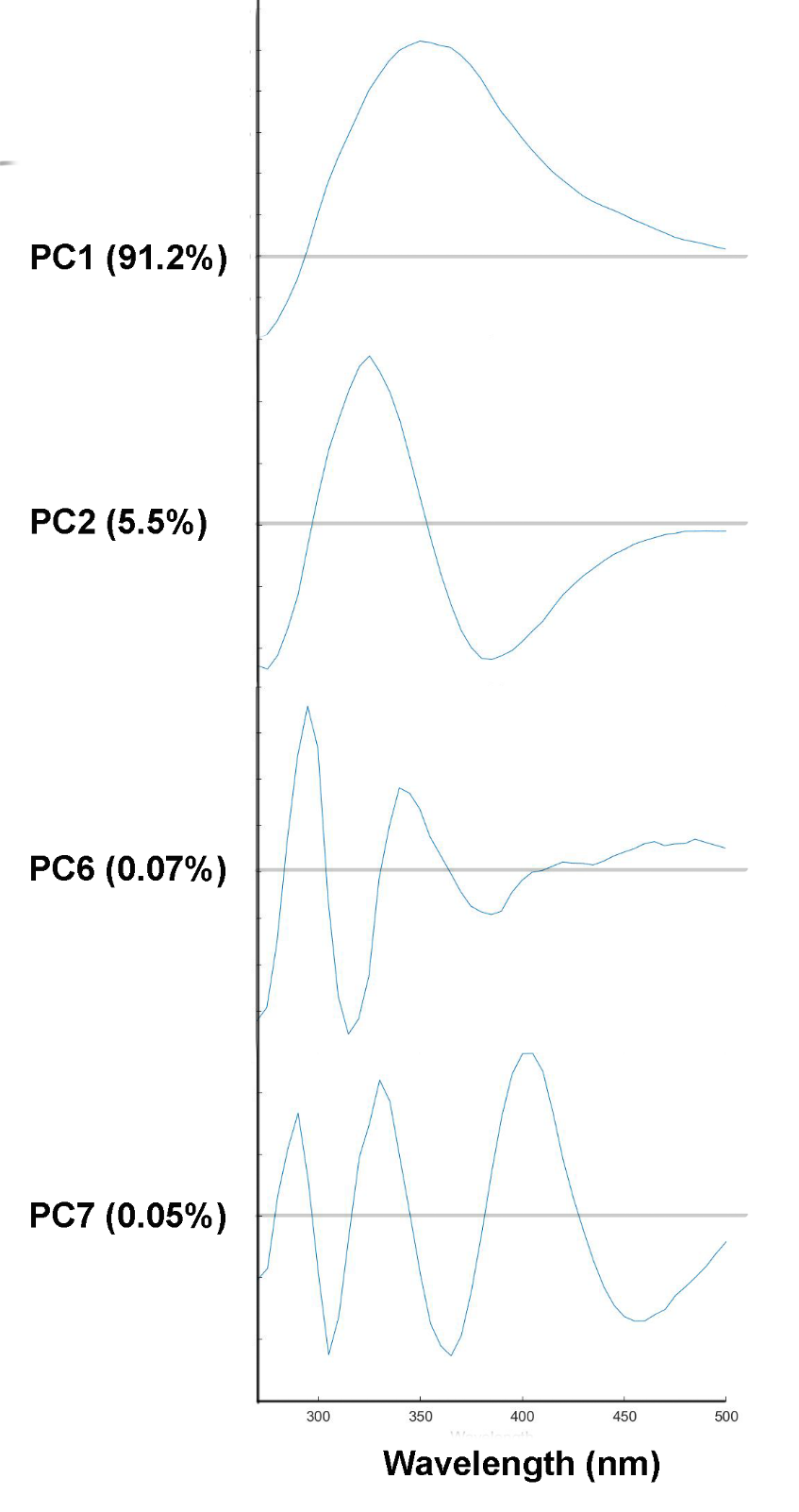


**Figure S2:** The factor loadings plots. Principal component (PC) 1, PC2, PC6 and PC7 summarized 97% of the PCA model.
